# The effect of nano-bio fusion gingival gel versus palatal stent on the palatal wound healing after harvesting free gingival graft: a randomized controlled clinical trial

**DOI:** 10.1038/s41405-025-00360-6

**Published:** 2025-08-11

**Authors:** Sara Mohamed Mahmoud Abdelrehim, Weam Ahmed Elbattawy, Omar Ahmed Mahmoud Ashour

**Affiliations:** Oral Medicine and Periodontology Department, Faculty of Dentistry, Cairo University, Cairo, Egypt

**Keywords:** Dental materials, Dentistry

## Abstract

**Introduction:**

This study aimed to compare two different approaches for palatal wound healing following free gingival graft (FGG) harvesting: one involving Nano Bio-Fusion (NBF) gingival gel used in conjunction with a palatal stent, and the other using a palatal stent alone. Outcomes were assessed in terms of wound healing, post-operative pain, and patient satisfaction.

**Methods:**

This parallel-grouped, two-arm, single-blinded, randomized controlled trial (RCT) included twenty-six patients with mucogingival defects that required harvesting an epithelialized free gingival graft (FGG). Patients were randomly allocated into either test group (NBF gingival gel and palatal stent; n = 13) or control group (palatal stent only; n = 13). Wound healing, the primary outcome, was evaluated over a 30-day period, while secondary outcomes included post-operative pain—measured using the Visual Analog Scale (VAS) and analgesic consumption—and patient satisfaction.

**Results:**

In the test group, wound healing showed statistically significant higher healing index score than control group after 3 days (*P* = 0.017), then no statistical significance was noted. Regarding post-operative pain, the test group showed statistically significantly lower pain scores (VAS) than control group in the first week, followed by no statistical significance in the second week. In the third day, the test group showed statistically significant lower analgesic consumption dose (*P* = 0.024) with overall statistically significant higher satisfaction score than control group (*P* = 0.002).

**Conclusion:**

Within the limitations of this study, the results suggest that NBF gingival gel may promote early-stage palatal wound healing, reduce postoperative pain and analgesic consumption during the first week, and enhance overall patient satisfaction.

**Clinical trial registration:**

(NCT05442359 | | https://www.clinicaltrials.gov/ 30-June-2022).

## Introduction

A key function of healthy keratinized gingival tissue is its role as a physical protective barrier against trauma such as that caused by tooth brushing. This protective function facilitates more predictable plaque control, ultimately contributing to the maintenance of gingival health around restored teeth and those undergoing orthodontic movement. Moreover, the preservation of periodontal health depends on the presence of an adequate zone of keratinized gingiva. Thus, keratinized tissue augmentation, whether in terms of thickness, height, or recession coverage around teeth or implants, is a common mucogingival concern that often necessitates harvesting free soft tissue grafts [1, 2].

Free soft tissue grafts, which are completely detached from their original site and placed onto a recipient bed, are considered the gold standard for increasing the width of keratinized tissue in areas with mucogingival defects due to their high predictability [3]. However, the need for a second surgical donor site which heals by secondary intention can lead to several complications, including postoperative pain, bleeding, extended surgical time, and aesthetically unfavorable outcomes at the recipient site due to discrepancies in tissue color and texture [4].

To address these complications, many modalities were proposed, including the use of palatal stents for wound protection [5, 6], platelet-rich fibrin [7], platelet concentrates [8], non-eugenol dressing (e.g., Coe-Pack) and collagen dressing [9], medicinal plant extract [5], hyaluronic acid [10], MEBO gel [11], ozonated oil [12] and low-intensity electrotherapy [13].

Over the past 15 years, nanotechnology has gained prominence across various fields, including dentistry. Nanotechnology facilitates the rapid and efficient cellular uptake of substances, thereby accelerating their biological effects. Nano Bio Fusion (NBF) gingival gel (not yet FDA approved) is a highly functional formulation that incorporates nano-antioxidants produced through nano-bio fusion technology. Its composition includes ultra-fine particles of vitamin C (0.2% ascorbic acid) and vitamin E (0.2% tocopherol acetate), along with 2% propolis extract, aloe vera, calendula, green tea extract, and additional ingredients such as sorbitol, cellulose, PEG-32, and glycerin which help regulate the formula’s pH and enhance its efficacy [14].

NBF gel has previously demonstrated antibacterial and anti-inflammatory properties, showing efficacy in managing peri-implant mucositis [15] and reducing pain after the surgical extraction of impacted lower third molars—likely due to its regenerative and antioxidant effects stemming from propolis and nano-vitamins C and E [16]. More recently, its application has been linked to the resolution of desquamative gingivitis and improvement in patient quality of life, with no reported side effects [17]. Based on these promising findings, the current study aimed to evaluate the effectiveness of NBF gingival gel, when applied to the palatal wound area and retained with a palatal stent, compared to the use of a palatal stent alone. The study assessed the gel’s impact on palatal wound healing, post-operative pain, and patient satisfaction following free gingival graft (FGG) harvesting from the palate.

## Methods

### Ethical review and registration

This randomized clinical trial was reviewed and approved by the Ethics Committee of Scientific Research at the Faculty of Dentistry, Cairo University in April 2022 (Approval No. 8422). It was also prospectively registered in the U.S. National Institutes of Health Clinical Trials Registry (Identifier: NCT05442359, registered on 30 June 2022). The study’s objectives were clearly explained to all participants, who provided written informed consent prior to enrollment. The trial was conducted in accordance with the ethical principles outlined in the Declaration of Helsinki for research involving human subjects, as revised in Fortaleza, Brazil (2013). Additionally, the trial was conducted and reported in compliance with the CONSORT guidelines.

### Study design and settings

This parallel-group study was designed as a single-blinded, two-arm randomized controlled clinical trial (RCT). Patient recruitment was conducted at the Periodontology Department, Faculty of Dentistry, Cairo University, Egypt. The study took place between July 2022 and July 2023, where patient recruitment and screening continued until the target sample size was reached.

### Sample size

The sample size calculation for this clinical trial was based on a previous study [18] that compared the effect of propylene mesh to custom-made acrylic palatal stent, using the palatal wound healing index after 30 days as the primary outcome. In that study, the mean healing index score and the standard deviation for the stent group was 1.9 ± 0.8756. Using alpha (α) level of (5%), β level of 0.8 (Power = 80%); the minimum accepted difference for independent samples t-test (d) was estimated as 1 and thus 11 participants were needed in each group to reach significance level. To account for a potential 20% dropout rate, the sample size was increased to 13 participants per group.The sample size was calculated using PS software version 3.1.2 (Vanderbilt University, Tennessee, USA).

### Participants

Patients were recruited from the Periodontology Department, Faculty of Dentistry, Cairo University, Egypt. All participants received both verbal and written explanations of the surgical procedure, including its risks, benefits, and timeline, and subsequently provided written informed consent. The inclusion criteria were as follows; (1) 18 years or older with mucogingival deformity that requires soft tissue augmentation by FGG; (2) systemically healthy individuals free of any systemic disease that would affect the healing outcome [19]; (3) palatal tissue thickness >2 mm assessed by University of Carolina (UNC) periodontal probe for bone sounding, placed perpendicular to the hard palate [20]; While the excluded patients were (1) smokers; (2) pregnant or lactating females; (3) patients with coagulation disorders such as a history of hemophilia, Von Willebrand disease or those under anticoagulant therapy [21]; (4) individuals with diseases known to alter the healing pattern as type 2 diabetes mellitus; (5) patients with reported allergy or hypersensitivity to any of the gel ingredients or the stent material used.

### Randomization

Twenty-six patients were randomly allocated into two equal groups: test group (NBF gel+ palatal stent) or control group (palatal stent only), using a computer-generated randomization list (www.random.org) with 1:1 allocation ratio. Allocation concealment was ensured using sequentially numbered, opaque, sealed envelopes, each containing the assigned treatment based on the randomization list. The randomization table was held by a non-recruiting faculty member (W.E.), who assigned the numbers to each patient. The sealed envelope containing the assigned treatment was opened after graft harvesting to maintain the purpose of concealment, and the treatment group was then disclosed to the operator (S.A.).

### Blinding

The healing scores in this trial were recorded by one calibrated examiner unaware of the group to which the participants were assigned. Since blinding the operator (S.A.) and the patients was not applicable, therefore this was a single-blinded randomized clinical trial in which only the outcome assessor (O.A.) was blinded to the group allocation.

### Preoperative phase

All patients received full mouth supragingival scaling and subgingival debridement using ultrasonic devices (Woodpecker UDS-K LED Ultrasonic scaler, China), followed by hand instrumentation with Gracey curettes (Nordent Gracey curettes, USA). Detailed oral hygiene instructions were also provided. Alginate impressions of the upper arch were taken to fabricate a customized palatal stent. The fit of the stent was checked prior to the surgical procedure, and any necessary adjustments were made to ensure proper adaptation for each patient.

### Surgical procedures

**Fig. 1:**
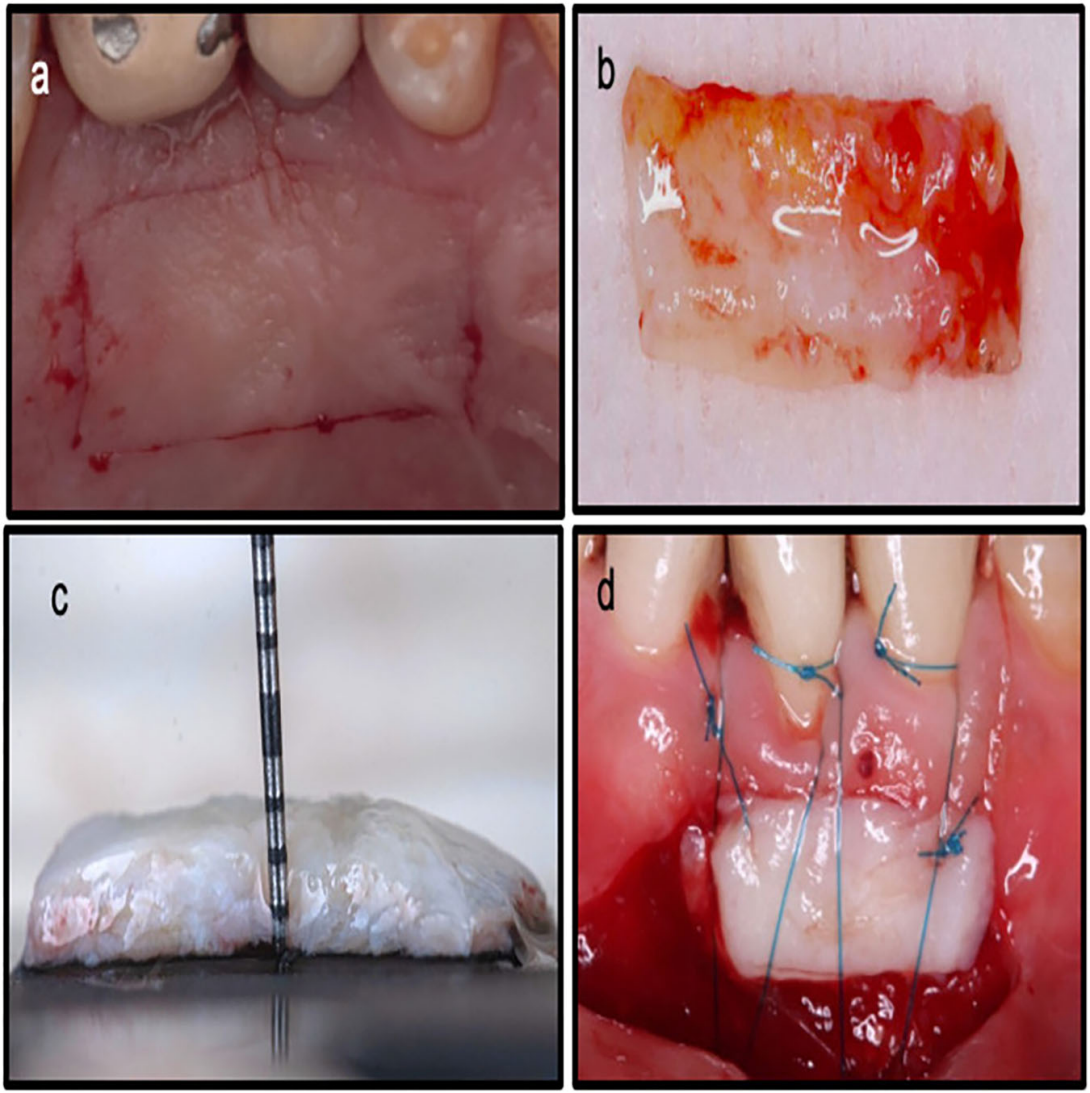
Steps for harvesting a free gingival graft from the palate. **a** Clinical photograph showing the primary incisions for FGG harvesting, **b** FGG harvested before removing the adipose tissue, **c** A periodontal probe used to ensure a uniform graft thickness, and **d** The FGG sutured to the recipient site for gingival augmentation.

The primary surgical site requiring a free soft tissue graft from the palate was prepared according to each individual case. The dimensions of the required graft were determined and transferred to a tin foil template in the desired shape and size, after which the FGG was harvested from the palate [22]. The palatal donor site was anesthetized via local infiltration using 4% articaine (Artinibsa Inibsa, Spain) with 1:100,000 epinephrine. The FGG was harvested according to the template size by placing two horizontal incisions using 15c blade, with the coronal incision made 1–2 mm apical to the gingival margin. Two vertical incisions were then made to delineate the harvesting area (Fig. 1a). The blade was inserted along the coronal horizontal incision at one edge, perpendicular to the bone. Once the desired graft thickness (1.5–2 mm) was achieved, the blade was angled parallel to the palate and moved in a mesio-distal direction, elevating the graft until fully detached. The harvested graft was placed on a sterile gauze moistened with saline to prevent shrinkage. Adipose tissue was removed to maintain uniform graft thickness (Fig. 1b, c). The graft was then used either epithelialized or de-epithelialized, depending on the intended purpose, and sutured at the recipient site (Fig. 1d).

**Fig. 2:**
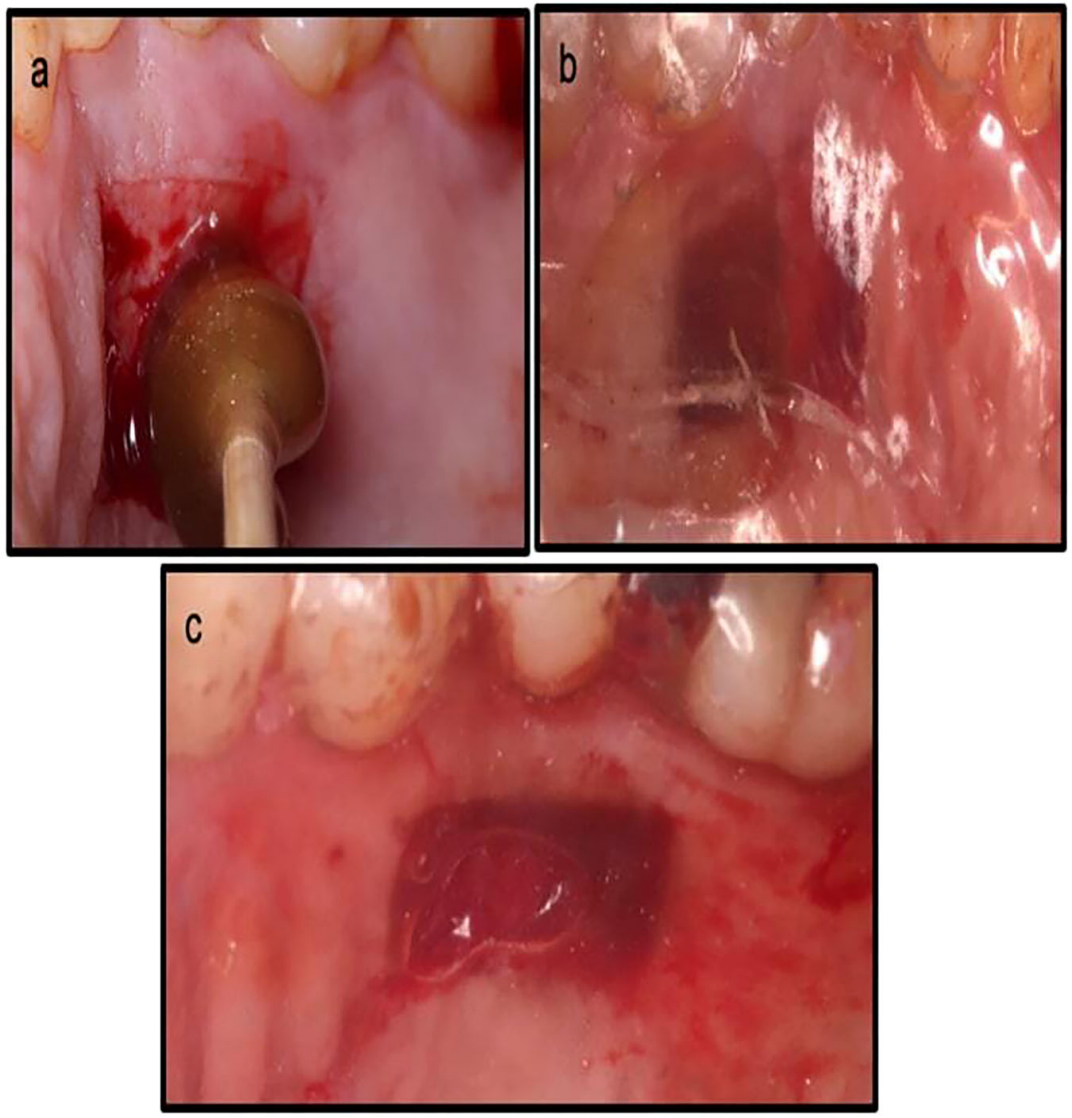
Palatal wound management in the test and control groups. **a** Photograph showing NBF gel application on the palatal wound, **b** Palatal stent covering the palate to keep the gel in place for the test group, wound size (width*length*thickness) (9.0*14*2.0). **c** Palatal stent covering the wound in the control group without gel application, wound size (width*length*thickness) (5.0*11*2.0).

Palatal bleeding was controlled by applying pressure with a sterile gauze for 5 minutes in both groups. In the test group, NBF gingiva gel (NanoCureTech. Gangdong-gu, Seoul, Korea) was applied to the donor site using a cotton pellet (Fig. 2a), and the gel was retained in place by the prefabricated palatal stent (Fig. 2b). Patients were instructed to reapply the gel 3 times daily for 4 weeks [17, 23], as complete epithelization is typically within 2-4 weeks [24, 25]. The stent was to be removed only during gel application and cleaning purposes, then worn during meals to prevent friction from food.

In the control group, the palatal wound was covered only with the palatal stent. Patients were instructed to wear the palatal stent full time for the first 2 days [20], and to begin removing it for cleaning purposes on the third day. Like the test group, they were instructed to wear the stent while eating to minimize friction from food particles (Fig. 2c). In both groups, the stent was used for a duration of 2 weeks.

### Postoperative care

Patients were instructed to consume soft diet during the first week and to avoid mechanical trauma. For the recipient site, postoperative instructions included avoiding tooth brushing and flossing around the surgical area for two weeks. Starting from the third week post-surgery, patients were advised to gently brush the surgical site using an ultra-soft tooth brush with the roll technique [22] and to rinse with 0.12% chlorhexidine mouth wash (Hexitol, The Arab Drug Company, Cairo, Egypt) twice daily for 2 weeks [10]. To control postoperative infection, systemic antibiotics (amoxicillin 500 mg/tid; GlaxoSmithKline, Cairo, Egypt) were prescribed for 5 days [22]. Analgesics (Biprofenid 150 mg; Sanofi Aventis, Cairo, Egypt) were prescribed for pain control as needed for 7 days [26]. Patients were instructed to count the number of analgesic pills consumed over a 7 day period [6], and to complete a questionnaire for 14 days, recording their VAS pain score prior to taking any analgesics [27]. At the end of the trial, they were also asked to fill another questionnaire to assess their overall satisfaction [28].

### Outcomes

**Fig. 3:**
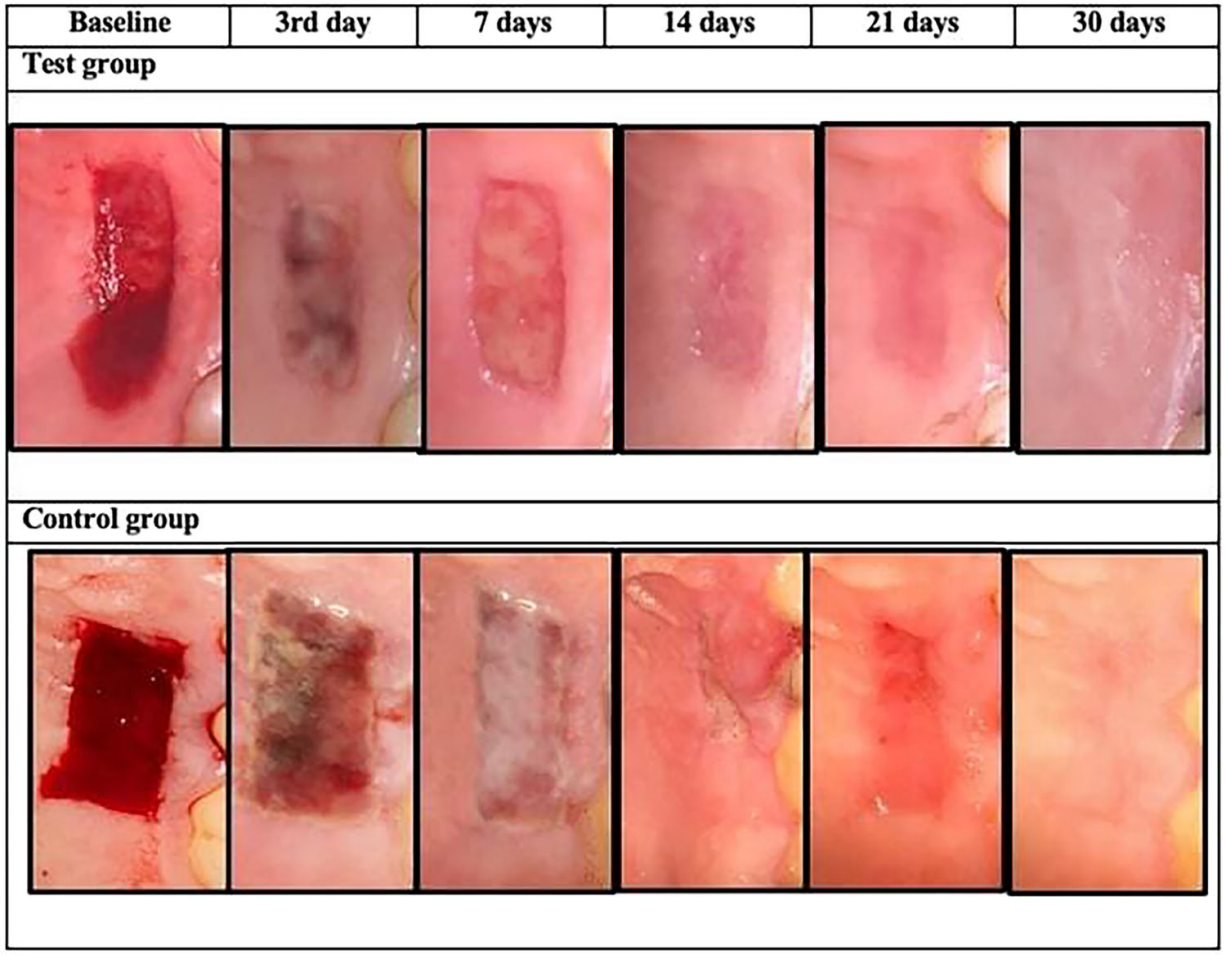
The figure shows phases of clinical healing patterns in test ad control groups from day 3 to day 30. A case from the test group with graft dimension (width*length*thickness) (5.0*10*1.5) clinical healing pattern from day 3 to  day 30. A case from the control group with graft dimension (width*length*thickness) (7.0*12*2.0) clinical healing pattern from day 3 to day 30.

Soft tissue healing of the palatal wound, assessed using the Landry Healing Index [29], was defined as the primary outcome. The Landry Healing Index was recorded on days 3, 7, 14, 21 and 30, with scores ranging from 1 to 5 (Fig. 3). Post-operative pain was evaluated using the VAS scale (0–10) over a period of 14 days, where “0” indicated “no pain” and “10” indicated “worst pain” [27]. The number of analgesics consumed was recorded by the patients daily for a total of 7 days [6, 30]. Patient satisfaction was assessed using a 3-item questionnaire addressing: their willingness to repeat the same procedure again, whether they would recommend this surgical procedure to others, and their overall satisfaction with the results. Responses were recorded on a 7-point scale, where 1 indicated “not at all likely (or not at all satisfied)” and 7 indicated “very likely (or very satisfied)” [28].

### Statistical analysis

Numerical data were explored for normality by checking the distribution of data and using tests of normality (Kolmogorov-Smirnov and Shapiro-Wilk tests). Age, graft width, length and thickness data showed normal (parametric) distribution while healing index, pain, analgesic dose and satisfaction scores data showed non-normal (non-parametric) distribution. Data were presented as mean, standard deviation (SD), median and range values. For parametric data; Student’s t-test was used to compare between mean age, graft width, length and thickness in the two groups. For non-parametric data; Mann-Whitney U test was used to compare between the two groups. Friedman’s test was used to study the changes by time within each group. Dunn’s test was used for pair-wise comparisons when Friedman’s test is significant. Qualitative data were presented as frequencies and percentages. Fisher’s Exact test was used to compare between the two groups. The significance level was set at P ≤ 0.05. Statistical analysis was performed with IBM SPSS Statistics for Windows, Version 23.0. Armonk, NY: IBM Corp.

## Results

**Fig. 4:**
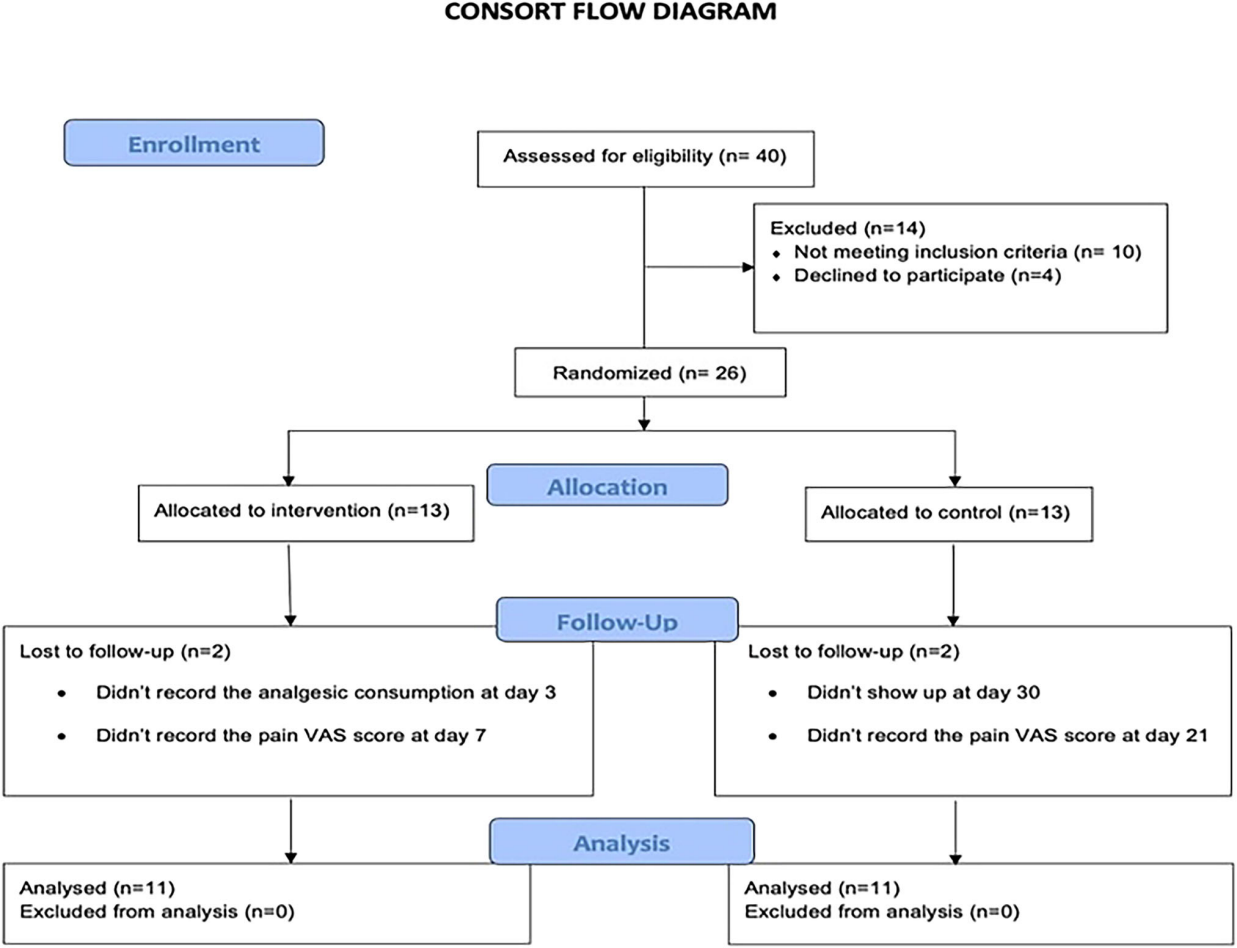
CONSORT flow chart.

This RCT included 26 patients (16 females and 10 males), aged 18-40 years old, who were scheduled for various periodontal and peri-implant plastic surgeries requiring harvesting of palatal mucosal grafts, either epithelialized or de-epithelialized. The patients were evenly divided into 2 groups: 13 in the test group (11 females and 2 males) and 13 in the control group (5 females and 8 males). A total of 4 patients (2 from each group) dropped out due to failure to attend follow-up sessions or non-compliance with the post-operative instructions (Fig. 4, CONSORT flow chart). Both groups were balanced in terms of age and gender. No statistically significant difference was observed between the two groups with respect to gender distribution, mean age, mean graft dimensions (length, width, thickness), and wound surface area (*P* > 0.05 / Table 1).

**Table 1:** Fisher’s Exact test and Student’s t-test for comparisons between baseline characteristics.

Patients’ data	Test group (NBF gel+ palatal stent) (*n* = 11)	Control group (Palatal stent) (*n* = 11)	*P* value
**Gender [*n*, (%)]**			
Male	2 (18.2%)	6 (54.5%)	0.183
Female	9 (81.8%)	5 (45.5%)	
**Age [Mean, SD]**	37 (6.7)	32.1 (7.5)	0.120
**Graft dimensions [Mean (SD), Median (IQR)]**			
Width (mm) [Mean (SD)]	6.8 (1.8)	5.6 (0.7)	0.054
Width (mm) [Median (IQR)]	6 (5.2–8.7)	6 (5–6)	
Length (mm) [Mean (SD)]	13 (3.9)	14.4 (3.7)	0.430
Length (mm) [Median (IQR)]	12 (10–15)	14 (12–15)	
Thickness (mm) [Mean (SD)]	1.9 (0.3)	1.9 (0.2)	0.697
Thickness (mm) [Median (IQR)]	2 (1.6–2)	2 (1.6–2)	
Wound area (mm^2^) [Mean (SD)]	87.9 (30.5)	80 (17.4)	0.4643
Wound area (mm^2^) [Median (IQR)]	84 (61.8–117)	84 (70.5–90)	

^*^Significant at *P* ≤ 0.05.

### Healing index score (Landry healing index)

There was a statistically significant increase in the healing index score over time within each group (*P* < 0.001). In the test group, a statistically significant increase in healing index score from 14 to 21 days (*P* < 0.001) was observed, while no statistically significant changes were noted from 3 to 7 days, 7 to 14 days, and from 21 to 30 days. In the control group, a statistically significant increase in the healing index score from day 3 to day 7, and from 14 to 21 days (*P* < 0.001) was noted. However, no statistically significant difference was observed from days 7 to 14 and from 21 to 30 days.

When comparing both groups, the test group showed a statistically significantly higher healing index score than the control group after 3 days (*P* = 0.017). There were no statistically significant differences observed between groups after 7, 14, 21 and 30 days (*P* = 0.116, 0.801, 0.655, 0.655, respectively, Table 2).

**Table 2:** Palatal healing score using Friedman’s test for intra-group comparison and Mann–Whitney test for intergroup comparison.

	Test group (NBF gel+ palatal stent) (*n* = 11)		Control group (Palatal stent) (*n* = 11)		*P* value	*Effect size (d)*
	Median (Range)	Mean ( ± SD)	Median (Range)	Mean ( ± SD)		
**Healing index score**						
3 days	3 (2, 4) ^B^	3.09 (0.7)	2 (1, 4) ^C^	2.27 (0.79)	0.017*	1.082
7 days	4 (3, 4) ^B^	3.55 (0.52)	3 (2, 4) ^B^	3.09 (0.7)	0.116	0.631
14 days	4 (2, 4) ^B^	3.55 (0.82)	4 (3, 4) ^B^	3.73 (0.47)	0.801	0.084
21 days	4 (4, 5) ^A^	4.27 (0.47)	4 (4, 5) ^A^	4.36 (0.5)	0.655	0.154
30 days	5 (4, 5) ^A^	4.64 (0.5)	5 (4, 5) ^A^	4.73 (0.47)	0.655	0.154
***P*-value**	<0.001*		<0.001*			
***Effect size (w)***	0.616		0.875			

^*^Significant at *P* ≤ 0.05, Different superscripts (A, B, C) in the same column indicate statistically significant change by time.

### Pain (VAS score)

Patients in the test group showed a statistically significant decrease in pain scores from the first day to day 7 (*P* < 0.001), followed by a non-statistically significant change from day 7 until the end of the follow up period. As for the control group, there was a statistically significant decrease in pain scores from the first day to day 9, after which non-statistically significant changes were noted from day 9 until the end of the follow up period (day 14). When comparing both groups, the test group showed statistically significant lower pain scores than the control group during the first week. Starting from day 8, no statistically significant difference in pain scores was found between the two groups. After the 10^th^ day, none of the cases in either group reported pain (Table 3).

**Table 3:** Descriptive statistics and results of pain scores using Mann-Whitney U test for inter-group comparison and Friedman’s test for intra-group changes.

Time	Test group (NBF gel+ palatal stent) (*n* = 11)		Control group (Palatal stent) (*n* = 11)		*P*-value	*Effect size (d)*
	Median (Range)	Mean (SD)	Median (Range)	Mean (SD)		
Pain (VAS score)						
1 day	6 (5, 9) ^A^	6.36 (1.43)	8 (6, 9) ^A^	8 (0.89)	0.011*	1.234
2 days	5 (4, 7) ^B^	5 (1.18)	7 (5, 9) ^B^	6.73 (1.35)	0.006*	1.377
3 days	4 (2, 7) ^C^	4.09 (1.81)	6 (3, 9) ^C^	5.91 (1.58)	0.023*	1.082
4 days	3 (1, 5) ^D^	3 (1.26)	5 (3, 10) ^D^	5.09 (1.76)	0.003*	1.536
5 days	2 (0, 5) ^E^	2.36 (1.57)	5 (1, 7) ^E^	4.36 (1.69)	0.015*	1.145
6 days	1 (0, 2) ^F^	1 (0.89)	3 (0, 6) ^F^	2.64 (2.11)	0.040*	0.880
7 days	0 (0, 2) ^G^	0.45 (0.82)	2 (0, 4) ^G^	1.73 (1.56)	0.037*	0.889
8 days	0 (0, 2) ^G^	0.18 (0.6)	0 (0, 3) ^H^	1.09 (1.38)	0.054	0.68
9 days	0 (0, 0) ^G^	0 (0)	0 (0, 3) ^I^	0.45 (0.93)	0.069	0.475
10 days	0 (0, 0) ^G^	0 (0)	0 (0, 0) ^I^	0 (0)	1	0
11 days	0 (0, 0) ^G^	0 (0)	0 (0, 0) ^I^	0 (0)	1	0
12 days	0 (0, 0) ^G^	0 (0)	0 (0, 0) ^I^	0 (0)	1	0
13 days	0 (0, 0) ^G^	0 (0)	0 (0, 0) ^I^	0 (0)	1	0
14 days	0 (0, 0) ^G^	0 (0)	0 (0, 0) ^I^	0 (0)	1	0
***P*-value**	<0.001*		<0.001*			
***Effect size (w)***	0.922		0.928			

^*^Significant *P* ≤ 0.05, Different superscripts (A, B, C, D, E, F, G, H, I) in same column indicate statistically significant change by time.

### Pain (analgesics consumption)

When the two groups were compared over the study period, no statistically significant difference was observed regarding analgesic consumption at all timepoints, except on the third day, where the test group showed a statistically significantly lower analgesic dose compared to the control group (*P* = 0.024, Table 4).

**Table 4:** Mann-Whitney U test for comparison between groups and Friedman’s test for the changes within each group.

Time	Test group (NBF gel+ palatal stent) (*n* = 11)		Control group (Palatal stent) (*n* = 11)		*P* value	*Effect size (d)*
	Median (Range)	Mean (±SD)	Median (Range)	Mean ( ± SD)		
Analgesic consumption dose (mg)						
1 day	300 (150, 300) ^A^	259.1 (70.1)	300 (150, 450) ^A^	313.6 (80.9)	0.110	0.552
2 days	150 (0, 300) ^B^	177.3 (112.6)	300 (150, 450) ^B^	272.7 (112.6)	0.083	0.73
3 days	0 (0, 300) ^C^	95.5 (121.4)	300 (0, 300) ^C^	218.2 (103.1)	0.024*	1.022
4 days	0 (0, 150) ^D^	68.2 (78.3)	150 (0, 450) ^D^	122.7 (147.2)	0.443	0.297
5 days	0 (0, 150) ^E^	27.3 (60.7)	0 (0, 300) ^E^	54.5 (101.1)	0.559	0.183
6 days	0 (0, 150) ^E^	13.6 (45.2)	0 (0, 300) ^E^	40.9 (97)	0.509	0.169
7 days	0 (0, 150) ^E^	13.6 (45.2)	0 (0, 300) ^E^	27.3 (90.5)	0.948	0.014
***P*-value**	<0.001*		<0.001*			
***Effect size (w)***	0.743		0.806			

^*^Significant at *P* ≤ 0.05, Different superscripts (A, B, C, D, E) in the same column indicate statistically significant change by time.

### Patients’ satisfaction

Based on the three item questionnaire used to assess the overall patient satisfaction, the test group (NBF gel+palatal stent) reported a statistically significantly higher satisfaction score than the control group (palatal stent only) (*P* = 0.002, Table 5).

**Table 5:** Descriptive statistics and results of Mann-Whitney U test for comparison between patients’ satisfaction scores in the two groups.

Test group (NBF gel+ palatal stent) (*n* = 11)		Control group (Palatal stent) (*n* = 11)		*P* value	*s*
Median (Range)	Mean (SD)	Median (Range)	Mean (SD)		
5 (4, 6)	4.91 (0.83)	4 (2, 5)	3.55 (0.82)	0.002*	1.593

^*^Significant at *P* ≤ 0.05.

## Discussion

Mucogingival defects are defined as any deviation from the normal relationship between the gingival margin and the mucogingival junction (MGJ). These abnormalities may include a lack of keratinized tissue, gingival recession, high frenum attachment and deep pockets extending beyond the MGJ [31]. FGG harvested from the palate is widely used to increase the zone of keratinized tissue, prevent recession, enhance esthetics, and reduce hypersensitivity [32]. However, harvesting FGG can be associated with complications such as postoperative pain, discomfort, bleeding, delayed wound healing, prolonged surgical time, infection, swelling and palatal sensory dysfunction [4, 33].

Various techniques have been proposed to cover the denuded palatal donor site and mitigate these complications. Despite numerous approaches, no single method has yet been established as the gold standard for managing palatal wounds after graft harvesting. Therefore, this clinical trial aimed to evaluate, for the first time, the effect of NBF gingival gel retained by a palatal stent as a dressing material following FGG harvesting.

The inflammatory process involves increased production of reactive oxygen species (ROS), which creates an imbalance between oxidants and antioxidants and results in oxidative stress. This stress can exert both systemic effects and localized impacts on the oral soft tissues [34, 35]. Given the widespread adoption of nanotechnology, the nano-based NBF gingival gel-formulated with nano-antioxidants-offers a promising therapeutic solution. This gel comprises 3 primary bio-compatible nano-emulsion components with antibacterial, anti-inflammatory, and antioxidative properties: 2% propolis extract, nano-vitamin C and nano-vitamin E. It is also characterized by its rapid and immediate absorption into the oral tissues due to its nano-sized particles [36].

The vitamin C component in NBF gingival gel directly activates phagocytes and acts as a co-factor in 8 enzymatic reactions essential for collagen synthesis. Notably, the nano-form of vitamin C in NBF gel is 2 times smaller and 110 times more potent in stimulating collagen synthesis than conventional vitamin C, making it more readily absorbed and efficacious [23]. Vitamin E, another potent antioxidant in the formulation, works synergistically with vitamin C to promote mucosal re-epithelization. It plays a critical role in preserving cell membrane integrity and in accelerating the epithelization process [37]. According to Popovska et al. [23], antioxidant-rich formulations are highly effective in soft tissue healing. Therefore, NBF gel was proposed as a suitable dressing for palatal wounds following FGG harvesting.

In addition, the propolis component provides antibacterial, antifungal, antiviral, anti-inflammatory and antioxidant properties due to the presence of flavonoids [38]. Its anti-inflammatory action includes inhibition of prostaglandin synthesis, enhancement of the immune response through phagocytic activation and cellular immunity, and acceleration of epithelial tissues recovery [14].

The adhesiveness of propolis, combined with nano-emulsion elements such as magnesium and sodium ascorbyl phosphate, helps form a nano-bioactive protective film. This feature addresses intraoral retention challenges by ensuring that the gel remains in contact with the soft tissues. Its ability to penetrate and be absorbed by gingival and intraoral tissues may provide nourishment, promote rejuvenation, offer protection and enhance healing [39].

In addition to the nano-formulated vitamins C, E and propolis, the NBF gingival gel also contains aloe vera, glycerin, and calendula. Aloe vera has shown significant clinical benefits in periodontal therapy by possessing antibacterial, antifungal, antiviral, antioxidant, and immunomodulatory properties [40]. Calendula is known for its wound-healing properties and can be used to aid oral wound healing. It possesses anti-inflammatory, antimicrobial, and antioxidant properties, which can promote faster healing and reduce discomfort in the mouth [41]. In addition, glycerin acts as a humectant, which helps retain moisture at the wound site and maintain gel consistency, which supports sustained therapeutic delivery [42]. Although the individual contributions of each component cannot be completely isolated in a multi-ingredient formulation, their synergistic interaction likely underlies the early wound healing, pain reduction, and higher patient satisfaction observed in the test group.

This clinical trial compared palatal wound healing outcomes between the test group (NBF gel + palatal stent) and the control group (palatal stent only). A statistically significant difference in healing index was observed at day 3 in favor of the test group. On that day, the test group achieved a healing score of (3), characterized by 25-50% redness, no bleeding on palpation, no granulation tissue, no suppuration and no exposed connective tissue. In contrast, the control group reached a healing score of (3) on day 7. This early improvement in the test group is likely due to the combined effect of ingredients that promote tissue repair. Propolis, aloe vera, and calendula all stimulate fibroblast activity, collagen production, and angiogenesis [40, 43, 44]. The anti-inflammatory and antimicrobial properties of aloe vera and calendula, mediated through flavonoids and polysaccharides, could have further contributed to an optimized healing environment [40, 44]. In addition, vitamin C and E support healing by promoting collagen cross-linking, protecting cells, and accelerating epithelial repair [37].

However, no statistically significant differences were noted between the groups from week 1 to week 4, suggesting that the effect of NBF gel was most evident during the early stages of healing. Comparatively, the wound healing outcomes with NBF gel appear similar to those reported for hyaluronic acid [45] and superior to those observed with Alvogyl [46].

The statistically significant reduction in post-operative pain scores observed in the NBF gingival gel group during the first week, compared to the control group, supports its effectiveness in promoting early wound healing. This improvement was evident in greater pain reduction and reduced analgesic consumption during the initial post-operative period. The highest reported pain score was 6 on day 1 in the test group compared to score 8 in the control group. Pain subsided by day 5 in the test group whereas it persisted until day 6 in the control group. These findings suggest that the analgesic effect of NBF gel is comparable to that of hyaluronic acid [45] and Alvogyl [30]. Notably, by day 7, NBF gel showed superior pain reduction compared to these materials, potentially due to multiple active ingredients. Propolis, calendula, and aloe vera all reduce pain by blocking COX-2 and prostaglandins [41, 47, 48]. Propolis also blocks NF-κB, while aloe vera blocks bradykinin, both playing a role in reducing nerve sensitivity [47, 48]. Vitamin C supports this by lowering inflammatory cytokines (IL-6, IL-8) and boosting antioxidant defense. Together these actions create less painful wound environment [49].

Although a significant reduction in pain scores was reported in the test group during the first post-operative week, both groups showed no statistically significant differences on days 8 and 9. From day 10 to day 14, no pain was reported in either group, reflecting the natural healing cascade which eventually took place. This trend was also reflected in analgesic use: the test group reported zero analgesic consumption by day 3, whereas the control group reached this point by day 5. These findings suggest that the active components of the gel may have contributed to a faster pain relief. Consequently, patients in the test group reported higher satisfaction scores than those in the control group, likely due to the gel’s soothing effect upon application.

The major limitation of this study is the lack of wound standardization, which should be addressed in future research to improve consistency across cases as this can affect healing time and pain perception. It was reported that analgesic consumption demonstrated a positive correlation with increased graft height [22], while a greater apico-coronal dimension was associated with increased postoperative pain [50]. Conversely, grafts with a width less than 14 mm were linked to reduced patient discomfort [51]. However, even though this study lacked wound standardization, there were no statistically significant differences in graft dimensions or wound surface area among the groups. This consistency in wound characteristics supports the reliability of our findings, allowing for an accurate interpretation of both pain and healing outcomes across the groups. Another significant limitation is the reliance on patient-reported outcomes. While these measures are valuable and reflect the actual patient experience, they are inherently subjective and can vary widely due to individual differences in pain perception. Additionally, the overall patient satisfaction and reported experience were not solely related to the palatal donor site, but rather reflected each individual’s overall experience of the surgical procedure. Although systemic conditions such as diabetes and smoking were excluded, healing responses still varied among patients. This is likely due to differences in healing capacity and pain thresholds, regardless of the post-operative technique used.

Furthermore, the study relied heavily on patient compliance and adherence to the gel application protocol. Each patient was given one tube of gel and reported its completion verbally by the end of the 30-day period. However, no objective method, such as compliance questionnaire or requesting the return of used gel tubes, was implemented to verify adherence. This remains a limitation and should be considered in future studies.

Although most patients reported a soothing sensation during gel application, a few reported application difficulties due to its sticky consistency. Additionally, patients in certain professions, such as teachers, experienced some discomfort while speaking as a result of the stent application. Finally, variability in individual pain thresholds and analgesic use made it challenging to discern whether pain relief was sought for the palatal donor site or the primary surgical area.

## Conclusions

Within the limitations of the current study, it can be concluded that: Post-surgical complications after FGG harvesting can be decreased using both a palatal stent and NBF gingival gel. NBF gingival gel seems to enhance the palatal wound healing, reduce the postoperative pain scores and the rate of analgesic consumption only at the early stages. However, this effect seems to disappear after the first week. Using NBF gel together with a palatal stent provided better overall patient satisfaction. These findings should be interpreted with caution, as the generalizability of the results is limited due to the small sample size, single-center study design, and reliance on patient-reported outcomes. Further multicenter studies with larger and more diverse populations are needed to validate and extend these results to broader clinical settings.

## Recommendations

Further clinical trials with a larger sample size might be needed to test and compare the effect of NBF gingival gel to other biomaterials that enhance the palatal wound healing in order to determine the actual effect of NBF gingival gel. Different healing indices could be used to evaluate the healing effect of NBF gel in addition to split-mouth design clinical trials that could overcome the variability in patient reported outcomes. Future studies should ensure wound standardization between groups to improve consistency and comparability. Objective measures to monitor patient adherence to the gel application protocol, either through a questionnaire or requesting patients to return used gel tubes, should be incorporated in future trials.

## Supplementary information

CONSORT CHECKLIST

## Data Availability

All data and materials used are available by the corresponding author on reasonable request.
